# The Influence of Very Low Illumination on the Postural Sway of Young and Elderly Adults

**DOI:** 10.1371/journal.pone.0103903

**Published:** 2014-08-01

**Authors:** Darja Rugelj, Gregor Gomišček, France Sevšek

**Affiliations:** 1 Biomechanical Laboratory, Faculty of Health Sciences, University of Ljubljana, Ljubljana, Slovenia; 2 Institute for Biophysics, Medical Faculty, University of Ljubljana, Ljubljana, Slovenia; University of California, Merced, United States of America

## Abstract

The purpose of the present study was to evaluate the influence of very low ambient illumination and complete darkness on the postural sway of young and elderly adults. Eighteen healthy young participants aged 23.8±1.5 years and 26 community-dwelling elderly aged 69.8±5.6 years were studied. Each participant performed four tests while standing on a force platform in the following conditions: in normal light (215 lx) with open eyes and with closed eyes, in very low illumination (0.25 lx) with open eyes, and in complete darkness with open eyes. The sequences of the tests in the altered visual conditions were determined by random blocs. Postural sway was assessed by means of the force platform measurements. The centre of pressure variables: the medio-lateral and antero-posterior path lengths, mean velocities, sway areas, and fractal dimensions were analysed. Very low illumination resulted in a statistically significant increase in postural sway in both the young and elderly groups compared to normal light, although the increase was significantly smaller than those observed in the eyes closed and complete darkness condition, and no significant effects of illumination on fractal dimensions were detected. The gains of the sways in the very low or no illumination conditions relative to the normal light condition were significantly larger in the group of young participants than in the group of elderly participants (up to 50% and 25%, respectively). However, the response patterns to changes in illumination were similar in the young and elderly participants, with the exception of the short-range fractal dimension of the medio-lateral sway. In conclusion, very low illumination resulted in increased postural sway compared to normal illumination; however, in the closed eye and complete darkness conditions, postural sway was significantly higher than in the very low illumination condition regardless of the age of the participants.

## Introduction

The human balance control system depends on important resources, such as cognitive processing, biomechanical constrains, age, and movement strategies [1], and is based on feedback from the somatosensory, vestibular and visual systems. The effect of the absence of visual information on balance can be assessed by the time for which a person is able to stand with their eyes closed [2] and can be measured as changes in postural steadiness via stabilometry [3], [4]. It has often been reported that, without visual information, postural steadiness significantly decreases [3], [4], [5], [6], [7]. This phenomenon has been documented in both young and older persons [7], [8], and postural sway is typically larger in elderly persons than in middle-aged and young persons [9], [10], [11], [8]. Dark environments thus affect adults in all age groups, although the effect is smaller in young adults than in middle-aged adults [12], and elderly persons are the most destabilised in conditions with no vision [12], [13], [11], [8].

However, different functional situations are present in environments with low and very low illumination. Such environments are commonly met indoors, for example, in corridors or in bedrooms and bathrooms at night [14]. These areas are considered to present the highest risk of falls for elderly people; it is estimated that approximately 20 to 55 per cent of all falls occur at home at night among the population that is 65 years or older [7], [15]. One of the possible reasons for this estimate is the fact that older people tend to rely more on the visual information for the postural stabilisation, and this effect increases with age [10]. Additionally, changes in the sensory context require a person to re-weight sensory flow to maintain stability [1]. Very low illumination is an example of such an environmental context in which sensory re-weighting is required. Subliminal pathologies and age-related changes in the somatosensory systems, including vision [11], contribute to decreased postural steadiness in the elderly. Individuals with specific sensory loss are therefore limited in their abilities to re-weight postural sensory dependence and are thus at higher risks of falling in particular sensory context [1]. It has been shown that the postural response of the elderly to low illumination can increase the risk for instability and falls [16]. Low levels of illumination and the optical patterns associated with specific surface layouts (e.g., escalators) have been identified as environmental factors that have detrimental effects on older people’s postural controls [6]. The rate of falls in elderly adults with poor vision is markedly higher than that among the elderly with normal vision [17].

There is no agreement between the studies that have investigated the influence of reduced levels of the ambient illumination on postural steadiness. Low illumination (between 1 and 3 lx) has been determined to have no influence on postural sway in a group of 9–10 year-old school children [18], [19]. However, for elderly populations, the reported results regarding the influence of very low ambient illumination are conflicting. Some authors have reported increases in postural sway in environments with very low illumination compared to those with normal illumination [3], [6], [13], while others were unable to find any significant increase in postural sway [20].

A review of the current and past literature did not identify any study that compared the responses to closed eyes, very low illumination and complete darkness between young and elderly persons. The early studies of the influences of various illumination conditions, including complete darkness, that were performed by Edwards [3] revealed, for example, a strong dependency on visual information input and the amount of illumination. However, this author did not allow the participants time to adapt to the transitions between different illumination conditions. Therefore, the results of this author might partially be attributable to the adaptation process, which has been found to last up to 30 seconds and to affect postural stability [7].

Thus, the main purpose of the present study was to investigate the effects of different levels of illumination on postural steadiness. Furthermore, we studied and compared these effects between a group of young and a group of elderly participants. To address these effects, we measured postural sway in four different experimental conditions: normal light with eyes open, normal light with closed eyes, very low illumination (dim light), and complete darkness with eyes open. We hypothesised that very low levels of illumination would affect postural stability and increase postural sway. We also hypothesised that younger participants would be less affected by changes in illumination (i.e., very low illumination) than would the older participants.

## Methods

### 1 Participants

The forty-four adults that participated in this study included 18 young healthy participants (12 female and 6 male) and 26 elderly community-dwelling participants (20 female and 6 male). The details of the participants are presented in Table 1. The exclusion criteria were the use of any medication known to compromise balance, diabetes, impaired vision and injuries to the lower extremities or spine. The participants were also required to express no fear of darkness or of small spaces (e.g., elevators). The study was approved by Slovenian National Medical Ethic Committee. Prior to any measurements, all participants read information about the test protocol, received additional verbal explanations when required and provided written informed consent.

**Table 1:** Descriptive data for the participants in the study.

	Number of participants	Mean ± SD	Minimum	Maximum
**Age (years)**				
Young	18	23.8±1.5	22	26
Elderly	26	69.8±5.6	60	82
**Body mass (kg)**				
Young	18	66.3±11.6	45	88
Elderly	26	72.3±13.5	49	105
**Body height (cm)**				
Young	18	169.9±0.1	155	181
Elderly	26	163.9±0.1	155	180

### 2 Experimental procedure

Each participant underwent five consecutive measurements, each of which lasted 70 seconds. During each measurement, the participant was instructed to stand as still as possible while barefoot and with their feet close together on the force platform, while their hands were hanging relaxed, and their gaze was focused on a dot that was pasted to the wall. During the first trial (1) in the normal lighting conditions (215 lx), the participant’s eyes were open. The following three trials were characterised as follows: (2) normal lighting conditions with closed eyes (215 lx), (3) a very low level of illumination (dim light) (0.25 lx) with open eyes, and (4) complete darkness with open eyes. The sequences of the trials with the altered visual conditions (trials 2, 3 and 4) were randomised by using random blocks.

In the trials in the normal lighting conditions with closed eyes, the participants were told to keep their eyes closed, and this requirement was additionally enforced by covering the eyes with opaque night glasses. In the very low level of illumination trials, the assistant turned off the main light, turned on the dimmed light, and let the participant adapt to the new lightening conditions for 30 seconds. The same procedure was followed for the complete darkness with open eye trials with the exception of turning the dim light on.

### 3 Illumination

The room was illuminated either by a regular ceiling lamp or by a desk lamp. The illumination of the desk lamp was adjustable via voltage regulation with a transformer (ISKRA TRN, Slovenia). The illuminance was determined with a luxmeter (Luxmeter Testo 545 Lux, Fc Germany) that was placed at the gaze fixation point on the wall. Because the lowest illuminance levels at the gaze fixation point were close or below the detection level of the luxmeter, these levels were determined based on illuminance measurements taken from a selected position in close proximity to the desk lamp. To determine the illuminance values in this experimental condition, the correlation between the two luminance values was determined at higher levels of illuminance and extrapolated to the experimental conditions. Thus, illuminance below the detection limit of the luxmeter and more precise determinations at levels close to the detection limit at the gaze fixation point of the participant were possible.

#### 3.1 Illumination characteristics

The characteristics of illumination of the room, i.e., increases, stability and magnitude, were determined by measuring the illuminance in close proximity to the desk lamp during the first 5 minutes after the lamp was turned on. These measurements were performed at 5-second intervals during the first 100 seconds and at 30-second intervals thereafter. The variability in the room illumination measurements was below 1%. Thus, the room illumination was determined to have good temporal stability, and no significant time dependency was noticeable over the time interval of 70 seconds. The final illuminance value had already been reached in the first 5 seconds.

### 4 Instrumentation and data processing

Stabilometry was used to assess the amount of postural sway during upright quiet standing. The centre of pressure (CoP) movement position data were collected at a 50 Hz sampling rate using a portable force platform (Kistler 9286 AA, Winthertur, Switzerland). The raw data were stored on the disk of a PC-type computer using Kistler’s BioWare program. The data were later uploaded to a server running the Linux (Fedora 18) operating system. Data analysis was performed with a web-based software that was specially developed for the stabilometric measurements in our laboratory [21]. After smoothing the acquired CoP positions with Gaussian averaging over 3 adjacent points, the first 10 seconds of each data series were excluded, and the remaining 60 seconds of the data series were used for the analysis. We excluded the first 10 seconds from the analysis to eliminate the possible consequences of the transitions between different illumination conditions and to avoid the transition between stepping on the force plate and steady standing [22], [13], [23]. From the resulting data, the time domain and fractal measures were calculated. A number of measures in both domains have been used to investigate postural steadiness in response to changing illuminance levels. In the time domain these measures are the mean values and standard deviations of the CoP displacements and velocities and the total path length, and the lengths of the medio-lateral and antero-posterior CoP movements. Additionally, the area and the outline shape of the measured stabilogram were determined, and the fractal dimensions were calculated from the time series of the CoP movements.

The procedures to determine the spatiotemporal parameters of the stabilogram and the outline and sway area have been described elsewhere [24]. Briefly, the antero-posterior (AP) and medio-lateral (ML) path lengths were calculated from the measured time series of the COP positions Y_i_ and X_i_ as follows:
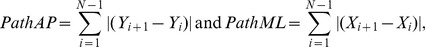
where the summation of the absolute values occurred over all N points. Similarly, the total CoP path length was determined as follows:



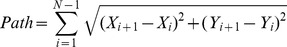



The mean velocity was obtained simply as Path/T, where T stands for the total measurement time of the N points.

To determine the sway area shape, the experimental data points were converted into polar coordinates relative to the data centre, and the points that were the furthest from the centre were determined in consecutive angular intervals. An analytic expression was fitted to these outline points [25] that determined the distance (R(φ)) from a chosen centre at a given angle φ via a Fourier series of N_max_ coefficients a_m_ and b_m_:
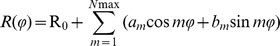



The origin of the coordinate system was then shifted to the centre of the calculated contour, and the procedure was repeated. Consequently, the shape of the smooth outline of the postural sway region is given by
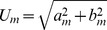
from which the surface area can be approximated as [26]:



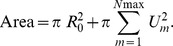



In addition to this area, the area of the elliptical region was calculated by principal component analysis (PCA) of the covariant matrix [27]. Here, the eigenvalues (σ_0_^2^) were calculated from the covariant matrix σ_xy_^2^

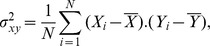
where 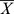 and 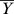 are the mean values, and the summation was performed over all N measured points.

The two eigenvalues are thus
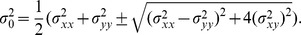



The sway area (Area_PCA_) may then by reproduced by an ellipse with the two principal axes 1.96 σ_0_. If the distribution is a bivariate Gaussian, such an ellipse includes 95% of the data points along each axis; consequently, 85.35% of all points lie within its perimeter [27].

The fractal dimensions of the ML and AP CoP positions time series were determined by the Higutchi method [28]. For a chosen time interval k, the length of the curve starting at m in the interval from 1 to k is determined as follows:
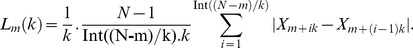



The length of the curve L(k) is then calculated by averaging over k values of m. If the curve in the given interval of k values is fractal, its length L(k) is proportional to k^−D^. The exponent D is the fractal dimension of the curve and can be easily determined by linear regression as the slope of the function ln(L(k)) versus ln(k). This procedure was tested by applying it to a time series that was generated as the sum of Gaussian noise in which the dimension was known to be 1.5 [28].

The following four sway parameters in the time domain were chosen for our analysis: (1) the mean CoP velocity during the 60 s measurement interval, (2) the medio-lateral and (3) antero-posterior path lengths, and (4) the sway area. The latter was calculated as the best area outline represented by the first N_max_ = 25 Fourier coefficients calculated from 100 angular intervals. In the fractal domain, the dimensions were calculated from the unfiltered ML and AP time series of the CoP positions for the two time intervals from 0 to 0.3 s and from 0.8 to 12 s. These short and long time intervals were chosen to exclude the transition region and to assure two predominantly linear relations of ln(L(k)) versus ln(k).

The calculation of the Romberg’s quotient was extended to encompass all of the illumination conditions. Thus, the gain for a variable in any particular condition was calculated as the quotient between that variable and the one obtained under the normal illumination with open eyes condition. The gains for time domain were calculated for both groups and for all variables.

### 5 Statistical analyses

The Statistical Package for Social Sciences (SPSS 20, SPSS Inc., Chicago, IL USA) was used for the statistical analyses. One-way repeated measures analyses of variance (ANOVAs) were performed to identify the effects of illumination and age on each of the dependent variables. Significant ANOVA findings were followed by LSD post hoc tests. To evaluate the differences between the age groups in the gains, t-tests for independent samples were used. The significance level was set at *p*<0.05.

## Results

### 1 Time domain variables

One-way repeated measures ANOVAs for the illumination conditions and age were calculated to compare the postural sway variables between the two groups of participants of different ages in each of the four different experimental conditions. Significant main effects of illumination condition were found for all four time domain variables. The calculated values for the mean CoP velocity was *F*_3,126_ = 25.852 (*p*<0.001), for the medio-lateral path length, *F*_3,126_ = 25.150 (*p* = 0.001), for the antero-posterior path length, *F*_3,126_ = 22.694 (*p* = 0.001), and for the sway area, *F*_3,126_ = 4.174 (*p* = 0.007). These findings indicate that there were effects of the amount of light on the postural sway variables. Significant main effects of age were also found for all of the analysed postural sway variables as follows: mean CoP velocity (***F***_1,42_ = 12.072, *p*<0.001), medio-lateral path length (*F*_1,42_ = 14.012, *p*<0.001), antero-posterior path length (*F*_1,42_ = 8.64, *p* = 0.007), and sway area (*F*_1,42_ = 7.484, *p* = 0.009). These findings indicate that age had effects on the postural sway variables. However, the interactions were not significant for any of the analysed postural sway variables: mean CoP velocity (*F*_3,126_ = 0.246, *p* = 0.864), medio-lateral path length (*F*_3,126_ = 0.591, *p* = 0.622), antero-posterior path length (*F*_3,126_ = 0.303, *p* = 0.823), and sway area (***F***_3,126_ = 0.551, *p = *0.648). These results indicate that there were not effects of group on postural sway in response to illumination.

The detailed results for all reported time domain CoP variables, which were separately determined from the experimental data recorded from 18 young and 26 elderly participants, are given in Table 2. From this table, it can be seen that the values for all four of the sway variables in the time domain were significantly higher during the initial experimental conditions (i.e., normal illumination with open eyes) in the elderly group than in the younger group. The same was true for all of the other experimental conditions (normal lighting conditions with closed eyes, very low level of illumination, and complete darkness with open eyes).

**Table 2:** Mean values for the time domain postural sway variables in the different illumination conditions for the groups of 18 young and 26 elderly participants (the bold figures indicate significant differences compared to the initial normal light eyes open condition).

	Mean velocity ± SD (cm/s)	ML path ± SD (cm)	AP path ± SD (cm)	Sway area ± SD (cm^2^)
**Normal light - open eyes**				
Young	1.11±0.23	46.38±8.82	38.25±10.1	3.34±1.16
Elderly	1.51±0.33	66.08±15.85	48.95±12.21	5.29±2.35
**Normal light - closed eyes**				
Young	**1.54±0.46**	**67.29±20.28**	**50.07±16.56**	4.84±2.12
Elderly	1.86±0.44***	80.76±18.89***	57.75±14.38**	5.57±1.91
**Very low illumination**				
Young	**1.31±0.36**	**56.22±13.95**	**43.02±14.98**	**3.99±1.77**
Elderly	1.68±0.42**	72.89±18.34**	55.28±14.92**	5.66±2.06**
**Complete darkness - open eyes**				
Young	**1.51±0.39**	**65.23±18.25**	**49.11±14.03**	4.69±1.96
Elderly	1.82±0.42***	78.23±18.52***	58.24±13.7***	5.08±2.59

ML - medio-lateral, AP - antero-posterior, SD - standard deviation, **p<0.01, ***p<0.001.

After finding no interaction of postural sway in response to illumination, we collapsed the groups and analysed the effects of different light conditions. LSD post hoc tests were performed to assess the differences between the four illumination conditions. We compared the normal illumination with open eyes to each of the following conditions: (1) closed eyes, (2) very low level of illumination, and (3) complete darkness with open eyes.

**Figure 1:**
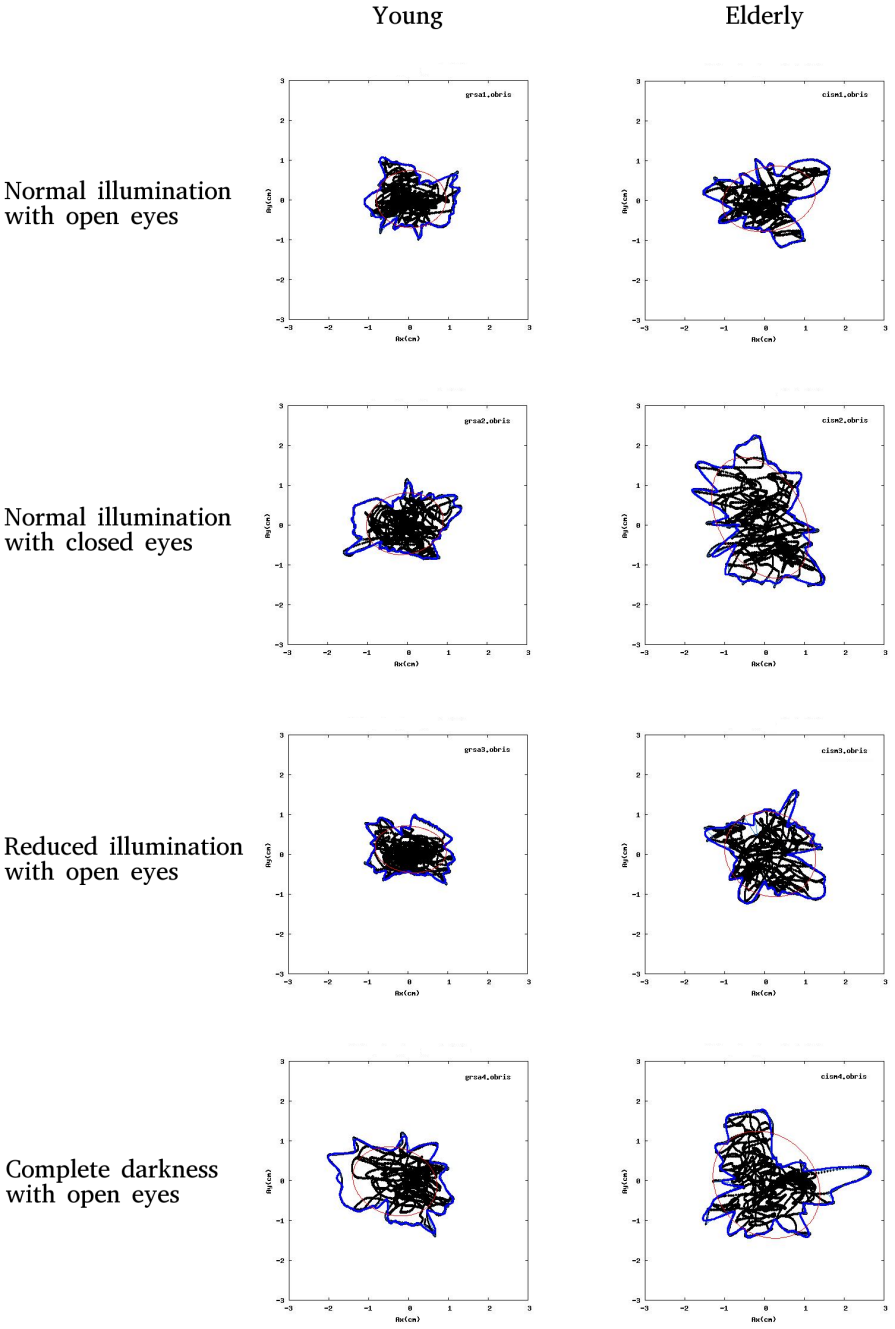
Typical CoP movement recordings. Typical CoP movement recordings from a young and an elderly participant under the four experimental conditions. The CoP path is indicated by the black dotted line, the CoP outline is indicated in blue, and the elliptic PCA region with 95% points along each axis is delineated by the red line.

The **mean CoP velocity**: An LSD post hoc test revealed a significant increase in the mean velocity for all of the altered light conditions compared to the normal light condition (*p*<0.001). When compared to the very low level of illumination, the increases in the mean CoP velocities in both no vision conditions were significantly elevated (*p*<0.001). The **medio-lateral and antero-posterior path lengths** increased in all of the altered light conditions relative to the normal light condition. LSD post hoc tests revealed that all of these differences were significant with *p* value that ranged from <0.001 to <0.003. Compared to the very low level of illumination, the increases in the medio-lateral and antero-posterior path lengths were both significantly higher in the no vision conditions (*p*<0.001). **Sway area:** LSD post hoc test revealed a significant increase of sway area for closed eyes and complete darkness compared to the normal light condition, p = 0.006 and p = 0.015, respectively. When compared to the very low level of illumination, the increases did not vary significantly between the very low level of illumination and any of the other light conditions; the LSD post hoc test results were *p* = 0.164, *p* = 0.088 and *p* = 0.087 for open eyes, closed eyes and complete darkness, respectively. The mean values and SDs of all of the described variables are given in the last two lines of Table 2. Typical movement recordings of the CoPs in the four experimental conditions for the young and elderly participants are shown in Figure 1.

### 2 Fractal domain variables

One-way repeated measures ANOVAs of illumination conditions and age were calculated to compare the postural sway variables in the fractal domain between the two groups of participants. The main effect of illumination condition was only significant for D_ML_(short) (*F*_3,126_ = 5.215 and p = 0.002), and the remaining fractal domain variables were not significant. The calculated value for D_ML_(long) was *F*_3,126_ = 2.332 (*p* = 0.077), for D_AP_(short) *F*_3,126_ = 2.710 (*p* = 0.048) and for D_AP_(long) *F*_3,126_ = 3.251 (*p* = 0.024). These results indicate that there were weak effects of the amount of light on the fractal domain variables. The main effects of the age were also non-significant for all of the analysed fractal domain variables: for D_ML_(short), *F*_1,42_ = 0.242, *p* = 0.625; for D_ML_(long), *F*_1,42_ = 0.419, *p* = 0.521; for D_AP_(short), *F*_1,42_ = 0.563, *p = *0.457; and for D_AP_(long), *F*_1,42_ = 1.411, *p* = 0.241. These findings indicate that there were no effects of age on the fractal domain variables of postural sway. The interactions were also not significant for any of the analysed fractal domain variables: for D_ML_(short), *F*_3,126_ = 1.805, *p* = 0.15; for D_ML_(long), *F*_3,126_ = 1.507, *p* = 0.216; for D_AP_(short), *F*_3,126_ = 1.579, *p* = 0.198; and for D_AP_(long), *F*_3,126_ = 1.996, *p* = 0.118. These findings indicate that there was no effect of age on postural sway in response to ambient illumination. The detailed results for all reported CoP variables in the fractal domain, which were separately determined from the experimental data recorded from the 18 young and 26 elderly participants, are given in Table 3.

**Table 3:** Fractal dimensions of the medio-lateral (D_ML_) and antero-posterior (D_AP_) CoP time series over short (0 s to 0.3 s) and long (0.8 s to 12 s) time intervals for the 18 young and 26 elderly participants.

	D_ML_ ± SD		D_AP_ ± SD	
	short	long	short	long
**Normal light - open eyes**				
Young	1.09±0.03	1.86±0.11	1.15±0.05	1.77±0.11
Elderly	1.09±0.02	1.89±0.11	1.13±0.04	1.85±0.09
**Normal light - closed eyes**				
Young	1.10±0.03	1.89±0.08	1.15±0.06	1.86±0.10
Elderly	1.10±0.04	1.91±0.10	1.14±0.05	1.86±0.12
**Very low illumination**				
Young	**1.09±0.03**	1.88±0.07	1.14±0.04	1.85±0.11
Elderly	**1.10±0.04** *	1.91±0.09	1.13±0.04	1.86±0.10
**Complete darkness - open eyes**				
Young	1.10±0.03	1.93±0.07	1.13±0.05	1.85±0.10
Elderly	1.10±0.03	1.91±0.10	1.13±0.04	1.87±0.10

*p<0.05.

### 3 Gain of the time domain variables

**Figure 2:**
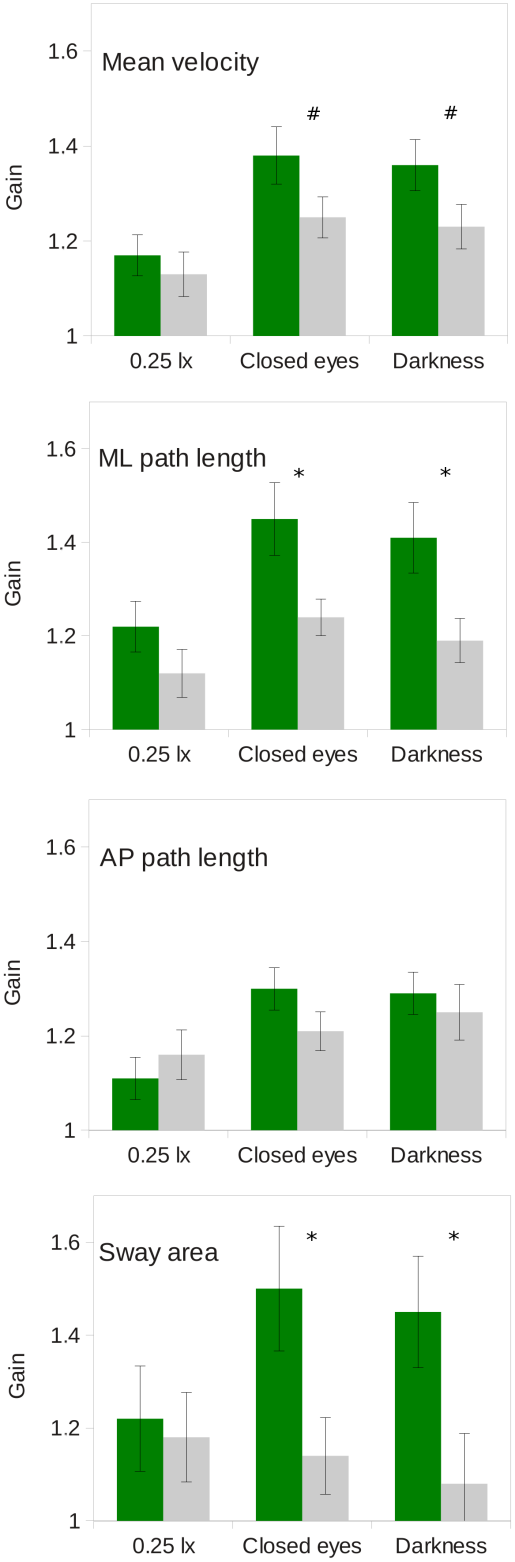
Gains in postural sway. Gains in postural sway expressed as the quotients between the normal light eyes opened conditions to the (1) very low illumination, (2) eyes closed and (3) total darkness conditions for the young participants (dark green) and the elderly participants (light grey). * indicates a significant difference between the two age groups at p<0.05, and # indicates marginal significance at 0.05<p<0.08.

The relative gains of the CoP variables were analysed to investigate the magnitudes of the differences between the conditions and between the groups. The gains were calculated separately for each age group and for all variables as the quotients relative to the value of the particular variable obtained under normal illumination with open eyes. Interestingly, in all of the evaluated conditions, the gains in the CoP fluctuations in the time domain were higher among the group of young participants compared to the elderly group. The gains of the elderly subjects were significantly smaller in the closed eyes condition in terms of the medio-lateral path length and sway area (t_42_ = 2.551, *p* = 0.015; t_42_ = 2.393, *p* = 0.021, respectively). The gain of the mean velocity was also smaller in the group of elderly participants; however, this difference was marginally significant (t_42_ = 1.827, *p* = 0.075). The same was also true for the complete darkness condition (medio-lateral path length t_42_ = 2.547, *p* = 0.015; sway area t_42_ = 2.292, *p* = 0.027, and mean velocity t_42_ = 1.820, *p* = 0.076). The highest relative difference was 1.50 and was observed in the increase in the sway area among the group of young participants when they were standing in normal light conditions with their eyes closed; the next largest difference (1.41) occurred in this group in the total darkness with open eyes condition (Figure 2).

## Discussion

The purpose of this study was to evaluate the influence of different environmental illumination conditions on the sways of groups of healthy young and elderly adults. The results revealed that both the young and elderly participants responded in a similar manner to changes in environmental illumination. Postural sway increased (i.e., postural steadiness decreased) the most in the closed eyes conditions, followed by the complete darkness with open eyes conditions. Postural sway was least affected by the very low level of environmental illumination. The responses to the very low level of environmental illumination were sways that were significantly larger than those in the normal light and significantly smaller than those in the no light conditions among both the young and elderly adults.

Compared to the eyes open normal illumination condition, the eyes open very low level of illumination condition yielded similar responses in both age groups. The illuminance in the dark room at the gaze fixation point was estimated to be 0.25 lx. This is the lowest level of illumination that has been reported (3 lx: [3], [13] and [20], 1 lx: [6]). It seems that the amount of light provided in this condition was not sufficient for accurate visual referencing and resulted in increases in postural sway. Therefore, we conclude that very low level of ambient illumination altered postural steadiness but significantly less so than did both of the no vision conditions. These results agree with those of Simoneau et al. [20] who reported a minimal effect of dim light on postural sway (from 2.6 to 11 per cent for different time domain variables) and the results of Brooke-Wavel et al. [6] who reported increased sway in dim light. The visual referencing differed between our study and that of Brooke-Wavel et al. [6] in terms of the spatial referencing markers; in our study, a single reference point was placed in front of the participants, while Brooke-Wavel et al. [6] used vertical lines to structure the environment. However, these authors did not report whether an adaptation period was allowed prior to the beginning of the measurements. Between these two studies, the ages of both groups were similar (present study: 69.8 years and Brooke-Wavel et al. [6]: 69.7 years). Our results indicate that in very low illumination, even when sufficient time is allowed for adaptation and an anchor point is provided, young and elderly adults exhibit increased postural sway, but these increases are less than those observed in both of the no vision conditions.

In the both the closed eyes and complete darkness no vision conditions, significant increases in postural sway were observed compared to the normal condition and the very low level of illumination condition in both age groups. These results agree with previous results regarding the absence of visual inflow in young [29] and elderly adults [30]. When the results between the two no vision conditions (i.e., normal light with closed eyes and complete darkness with open eyes) were compared within each group, no significant differences were found. Therefore, the hypothesis proposed by Rougier [31] that nearly all postural sway parameters are systematically higher in no light conditions with closed eyes compared to no light conditions with open eyes might indicate that the eyelid opening-closing mechanism can interfere with postural control. Rougier [31] reported a difference in stabilogram properties between conditions of standing with eyes closed in normal light and with eyes open in darkness. However, preliminary results from our laboratory [32] have shown that participants sway in complete darkness to similar extent regardless of whether their eyes are open or closed. Additionally, the results from both of the darkness conditions did not differ from those observed in the eyes closed in normal light condition. An advantage of our experimental arrangement over that of Rougier [31] might be the use of the night goggles that allowed the research assistant to continuously ensure the closure of the eyes in the dark room.

Age had no effect on the responses to the level of environmental illumination; the same patterns were observed in the young and the old groups. A similar finding was reported by Kinsella-Shaw et al. [13]. However, the absolute values were significantly higher for the older group, which agrees with previous results of studies investigating the postural sways of young and elderly adults [6], [30], [33]. It is also interesting to note that neither age nor illumination condition resulted in any significant changes in the fractal dimensions as determined from the medio-lateral and antero-posterior CoP time series over short (0 s to 0.3 s) and long (0.8 s to 12 s) time intervals with the exception of the effect of illumination on D_ML_(short). The insensitivity of the fractal dimensions to different illumination conditions agrees with the results of Stambolieva [34], who did not find any differences between open and closed eye conditions using nonlinear techniques; however, Doyle et al. [35] reported changes in antero-posterior fractal dimensions between open and closed eye conditions among elderly people using the same method reported here. This discrepancy might be because these authors used different time intervals for their dimension calculations and did not treat the short and long time intervals separately. The latter difference might also explain the generally smaller values for the calculated dimensions reported by these authors.

When the gains of the postural sway variables relative to the normal conditions (i.e., the normal illumination with open eyes condition) were compared between the two studied groups, considerably higher gains in all of the postural sway variables were observed in the young participants. Our results from the young participants revealed medio-lateral path length gains of 22 per cent gain in low illumination and 41 per cent in complete darkness. In contrast, the relative gains of the elderly participants were substantially smaller; 12 per cent was observed in very low illumination, and 19 per cent was observed in total darkness. These detected gains are significantly lower than those reported in the study by Edwards [3] who described a 32 per cent gain in postural sway in dim light and a 93.7 per cent gain in total darkness among a group of young participants. These differences might be explained by the lack of adaptation time to changes in changed illumination. Hence, a portion of the increase in postural sway might have been due to the initial phase of adaptation to changes in illumination during which higher postural sway variables could be expected. Specifically, Sozzi et al. [36] reported significant shifts in CoP position due to changes in visual input. The adaptation to darkness and decreased light requires considerable time [37], and during adulthood, the adaptation process slows with increasing age [38]. In our study, sufficient time was allowed between trials for the participants to adapt to the changes in illumination. Our results support the results and the conclusion of Johnson et al. [7] that sufficient time is needed to stabilise posture when sitting up in bed in dimly lit environments before any given action can be safely performed. However, some caution is needed when comparing these results because the participants in Johnson’s study had to address changes in two conditions; changing body position (from lying to a sitting position) in which orthostatic function plays an important role, and adaptation to low levels of environmental illumination. Moreover, in more dynamic conditions, such as gait, the transition between normal and dim light rather than the low level of light itself is the most destabilising effect regardless of gait speed [39].

Smaller gains in postural sway among elderly adults as expressed as Romberg quotients were reported by Prieto et al. [11] for the time domain variables, sway area and fractal dimensions. The Romberg quotients for the elderly adults reported in this study are even smaller. The only variable that increased more among the elderly adults than among the younger adults was the Romberg quotient for the mean velocity [11]. Why would young participants respond with higher relative gains in postural sway compared to the elderly participants when their eyes were closed or they were in complete darkness? We hypothesise that the sway of the young participants is, in normal conditions, well within the limits of stability, and although their sway increases more with decreased visual flow, it remains well within those limits. As reported by Sozzi et al. [36], in eyes closed conditions, young adults use 3% of their supporting surface, while elderly adults already use a larger support area for swaying. The perceptions of the slow velocity shifts in CoP or CoG are based on information coming from the visual and proprioceptive systems, while the vestibular system responds to higher velocity movements [40]. Therefore, it seems to be reasonable to assume that elderly persons adopt a strategy to avoid the high CoP velocities that results in increased postural sway. Thus, to prevent this increase in sway, older adults adapt to no-vision conditions with increased stiffness. This response of the elderly participants likely results from their previous experiences that are an important component for the selection of a strategy for a specific postural response [1] and on the reference of their own perceived stability borders that are a function of their egocentric reference frame [41]. This reference frame is based on information coming from the visual, proprioceptive and vestibular systems [42]. Additionally, increased activity of the ankle muscles in the no-vision conditions has been reported for young adults [36], and a similar phenomenon likely occurs in the elderly.

Thus, the main limitation of this study regarding its practical implications seems to be that the results are valid only for static situations, whereas in functional situations, the gaze moves within the visual field, which requires additional mental processing.

## Conclusion

Based on the analyses of the results of experiment, we accept our first working hypothesis and reject the second hypothesis. Very low illumination increased postural sway regardless of the age of the participants. Moreover, the gains exhibited in the postural sway measures by the young participants in altered illumination conditions were higher than those of the elderly participants across all of the time domain variables; however, the patterns of responses to the illumination changes were similar in both age groups. The higher gains among the young group might be interpreted as a hallmark of normal balance performance [43].
